# Persistent Hepatitis B Viral Replication in a FVB/N Mouse Model: Impact of Host and Viral Factors

**DOI:** 10.1371/journal.pone.0036984

**Published:** 2012-05-16

**Authors:** Shih-Hui Chen, Hui-Lin Wu, Jia-Horng Kao, Lih-Hwa Hwang

**Affiliations:** 1 Graduate Institute of Microbiology, National Taiwan University College of Medicine, Taipei, Taiwan; 2 Institute of Microbiology and Immunology, National Yang-Ming University, Taipei, Taiwan; 3 VYM Genome Research Center, National Yang-Ming University, Taipei, Taiwan; 4 Hepatitis Research Center, National Taiwan University Hospital, Taipei, Taiwan; 5 Graduate Institute of Clinical Medicine, National Taiwan University College of Medicine, Taipei, Taiwan; 6 Department of Internal Medicine, National Taiwan University Hospital, Taipei, Taiwan; Yonsei University, Republic of Korea

## Abstract

The mechanism underlying the chronicity of hepatitis B virus (HBV) infection has long been an interesting question. However, this mechanism remains unclear largely due to the lack of an animal model that can support persistent HBV replication and allow for the investigation of the relevant immune responses. In this study, we used hydrodynamic injection to introduce HBV replicon DNA into the livers of three different mouse strains: BALB/c, C57BL/6, and FVB/N. Interestingly, we found that an HBV clone persistently replicated in the livers of FVB/N mice for up to 50 weeks but was rapidly cleared from the livers of BALB/c and C57BL/6 mice. Flow cytometric analysis and quantitative reverse transcription PCR analysis of the mouse livers indicated that after DNA injection, FVB/N mice had few intrahepatic activated cytotoxic T lymphocytes (CTLs) and produced low levels of alanine aminotransferase, interferon (IFN)-γ, tumor necrosis factor (TNF)-α, and the CXCL9 and CXCL10 chemokines. These findings were in sharp contrast with those observed in BALB/c and C57BL/6 mice, reflecting a strong correlation between the degree of liver inflammation and viral clearance. Mutational analysis further demonstrated that a change of Asn-214 to Ser-214 in the HBV surface antigen rendered the persistent HBV clone clearable in FVB/N mice, which was accompanied by increased levels of activated CTL and upregulated expression of IFN-γ, CXCL9, and CXCL10 in the livers. These results indicate that the heterogeneity of the host factors and viral sequences may influence the immune responses against HBV. An inadequate activation of immune or inflammatory responses can lead to persistent HBV replication *in vivo*.

## Introduction

With an estimated 350 million people worldwide chronically infected, hepatitis B virus (HBV) infection remains a major health problem. More than 95% of acutely infected adults recover from the infection, whereas most of the neonatally transmitted infections become persistent [1], [2]. Chronic HBV infection can lead to hepatic cirrhosis and hepatocellular carcinoma (HCC) [3]. Although a prophylactic vaccine against HBV is currently available, HBV carriers who already have viral DNA in their livers still run a great risk of developing HCC. An investigation of the mechanisms underlying HBV persistence *in vivo* may lead to new approaches for treating and preventing the progression of chronic hepatitis B to life-threatening liver diseases.

The genetic background of the host and viral factors are believed to contribute to the different outcomes of HBV infection. Genetic polymorphisms of several host factors have been implicated in the susceptibility to chronic HBV infection, including estrogen receptor [4], killer cell immunoglobulin-like receptor (KIR) [5], interleukin 10 promoter [6], interferon-γ (IFN-γ) [7], tumor necrosis factor-alpha (TNF-α) promoter [8]–[10], and human leukocyte antigen (HLA) class II molecules [11], [12]. Among these factors, IFN-γ and TNF-α are two cytokines that can inhibit HBV replication non-cytopathically [13]–[16]. The genetic variations leading to low levels of IFN-γ and TNF-α production are associated with chronic HBV infection [7]–[10]. In addition, a recent genome-wide study has shown the HLA-DP loci belonging to HLA class II molecules to also be associated with chronic HBV infection, most likely due to a weaker CD4 T-cell helper response induced by these HLA molecules [17].

In addition to host factors, several viral factors have been reported to affect the innate or adaptive immune responses against HBV infection. The hepatitis e antigen (HBeAg) is a viral immunomodulatory protein that, via deletion or anergy, inhibits the HBV core (HBcAg)/HBeAg cross-reactive T-cell response [18]. The soluble hepatitis B surface antigen (HBsAg) significantly exhausts HBsAg-specific T-cell responses [19]. HBV polymerase blocks pattern-recognition receptor signaling by disrupting the interaction between IKKε and DDX3, a DEAD box RNA helicase [20]. Furthermore, it is likely that sequence diversity between different HBV genotypes or different HBV strains may influence the existence of particular epitopes, thus resulting in different immune response profiles [21]–[23].

An easily generated immunocompetent animal model is instrumental to the systematic investigation of host and/or viral factors pertinent to HBV persistence. Although mice cannot be infected by wild-type HBV, mouse hepatocytes can support HBV replication and produce infectious virions when viral DNA is directly delivered into the cells. Therefore, a genetically well-characterized inbred mouse should be an ideal model to study the mechanism of HBV persistence if HBV DNA can be efficiently and appropriately delivered to the mouse liver. We have previously employed hydrodynamic injection to deliver HBV replicon DNA, cloned in a pGEM4Z plasmid vector, into BALB/c, C57BL/6, and FVB/N mice. Surprisingly and interestingly, persistent HBV replication was maintained in FVB/N mice for up to 50 weeks but was rapidly diminished in BALB/c and C57BL/6 mice. Thus, we used these mouse strains to further investigate the host and viral factors pertaining to HBV persistence. In this study, we provide data demonstrating that mouse strains that elicit strong cytotoxic T lymphocyte (CTL) responses and induce strong inflammatory responses, e.g., BALB/c and C57BL/6, can clear HBV rapidly, whereas mice that induce low levels of CTL and weak inflammatory responses, e.g., FVB/N mice, tend to develop a persistent infection. In addition, we show that a single amino acid difference in the HBV surface protein can affect the activation of CTL responses and result in different rates of viral persistence.

## Results

### Host genetic backgrounds influence HBV persistence *in vivo*

**Figure 1:**
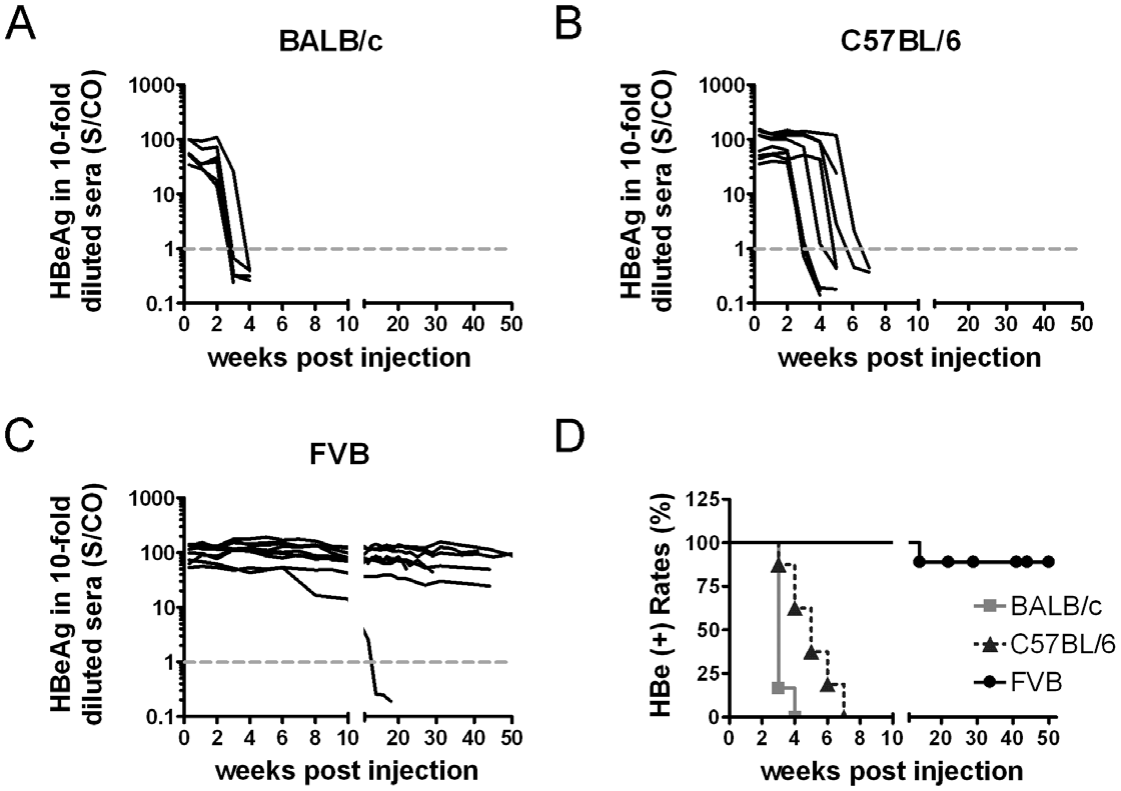
Long-term follow up of serum HBeAg in the animals injected with the pHBV1.3-B6 replicon DNA. Serum HBeAg was monitored regularly following hydrodynamic injection of pHBV1.3-B6 replicon DNA (10 µg/mouse) in BALB/c (A), C57BL/6 (B), and FVB/N (C) mice. Each line represents one animal. The levels of HBeAg in 10-fold diluted sera are shown as S/CO, signal-to-control ratio. The dotted gray lines represent the cut-off value of HBeAg. (D) The kinetics of HBeAg clearance in the three mouse strains over a period of 50 weeks are shown and were compared using the logrank test. The HBeAg-positive rates are as follows: BALB/c<C57BL/6 (**P<0.01), BALB/c<FVB/N (***P<0.001), and C57BL/6<FVB/N (***P<0.001).

We first examined how host backgrounds might affect HBV persistence *in vivo*. BALB/c (H-2^d^), C57BL/6 (H-2^b^), and FVB/N (H-2^q^) mice were hydrodynamically injected with a pHBV1.3-B6 replicon DNA that contains a 1.3-fold overlength of a genotype B HBV genome (Fig. S1). To mimic the quasispecies status of HBV in clinical settings, the B6 replicon DNA actually consists of six sub-clones derived from the same chronic hepatitis B patient (see the Materials and Methods). After DNA injection, serum HBeAg was used as a surrogate marker to monitor HBV persistence *in vivo*. The results revealed HBeAg to be rapidly cleared from the sera of BALB/c (Fig. 1A) and C57BL/6 mice (Fig. 1B) within four and eight weeks, respectively. In contrast, serum HBeAg persisted in most of the FVB/N mice for up to 50 weeks (Fig. 1C) and had cleared in only 1 out of 9 mice at 14 weeks post injection (wpi). The HBeAg-positive rates in FVB/N mice were statistically significant compared to those in BALB/c and C57BL/6 mice (Fig. 1D).

**Figure 2:**
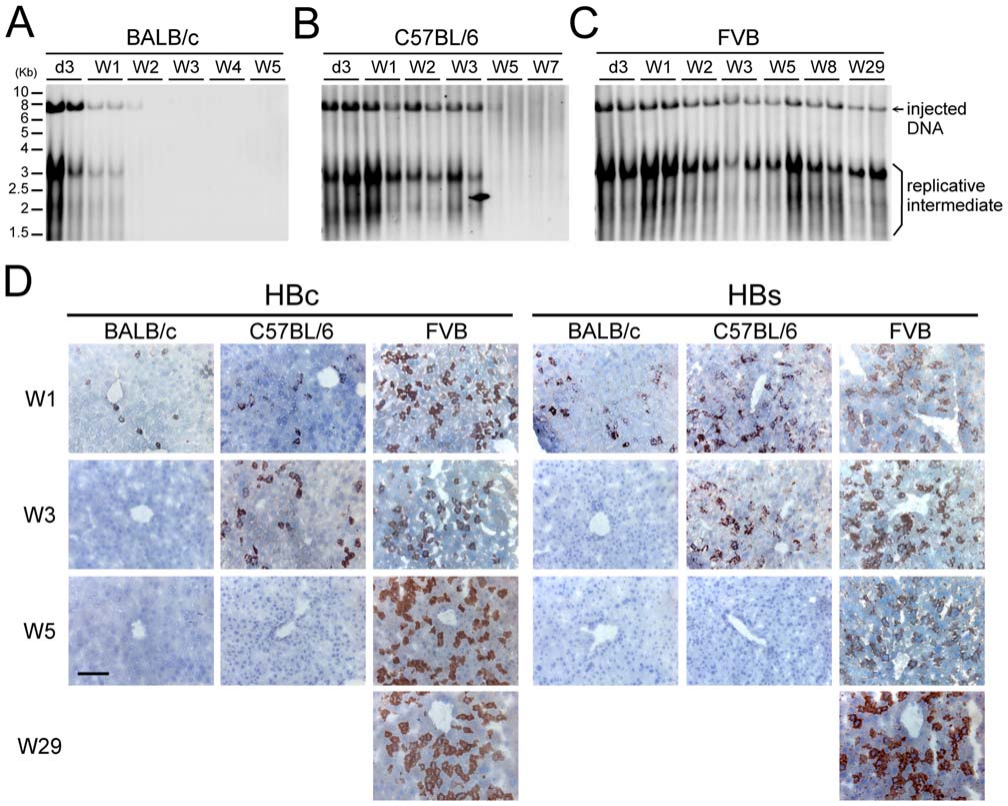
The duration of HBV replicative intermediates present in the livers of the replicon DNA-injected animals. BALB/c (A), C57BL/6 (B), and FVB/N (C) mice were hydrodynamically injected with 10 µg of the pHBV1.3-B6 replicon DNA. At the indicated time points, two animals were sacrificed. The liver genomic DNA was extracted and digested with *Hind*III (a single cut in the HBV replicon DNA), and then analyzed using Southern blot analysis with an HBV-specific probe. Each lane represents one animal. The sizes corresponding to the injected DNA, and the replicative intermediates are indicated on the right. (D) The IHC staining of HBcAg and HBsAg in the livers of the BALB/c, C57BL/6, and FVB/N mice injected with pHBV1.3-B6 DNA. Livers were harvested at the time points indicated. IHC staining was performed using anti-HBc or anti-HBs antibody on frozen sections. The scale bar represents 100 µm. The images are displayed at 200× magnification.

To ascertain the persistence of HBV in FVB/N mice, Southern blot analysis and immunohistochemical (IHC) staining were performed on the livers. HBV replicative intermediates and the episomal form of the injected DNA were detected in the livers of BALB/c mice injected with the pHBV1.3-B6 replicon DNA for up to two weeks (Fig. 2A) and in the livers of C57BL/6 mice for up to five weeks (Fig. 2B) versus up to 29 weeks in the livers of FVB/N mice (Fig. 2C). IHC staining of the liver sections also showed that HBcAg and HBsAg were persistently expressed in FVB/N mice but were rapidly cleared in BALB/c and C57BL/6 mice (Fig. 2D). Cytoplasmic HBcAg has been reported to be an indicator of HBV replication [24]–[26], whereas nuclear HBcAg is a stable species in the absence of HBV replication [27], [28]. Thus, the cytoplasmic staining pattern of HBcAg in the livers of FVB/N mice (Fig. 2D) strongly supports the replicative status of the HBV genome in these mice at week 29. Collectively, these results demonstrate that the pHBV1.3-B6 replicon DNA replicates persistently in FVB/N mice but not in BALB/c and C57BL/6 mice.

### HBeAg clearance correlates with elevated levels of alanine aminotransferase (ALT), increased activated CTLs, and the severity of inflammation in the livers

**Figure 3:**
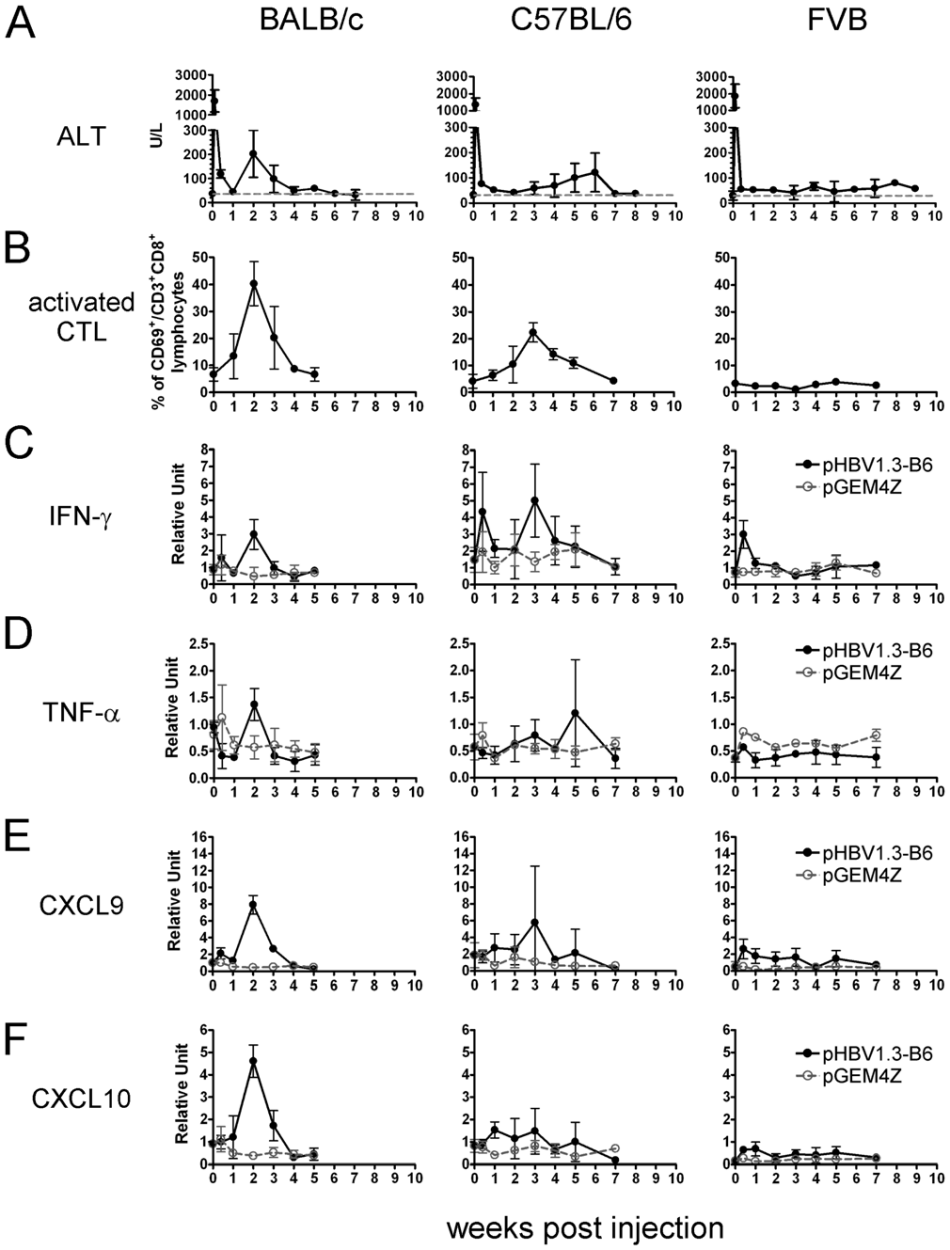
The kinetics of ALT elevation, infiltration of activated CTLs, and the expression of cytokines and chemokines in different mouse strains after DNA injection. Mice were injected with pHBV1.3-B6 DNA (10 µg/mouse), and three animals were sacrificed at the indicated time points after DNA injection. (A) The serum ALT levels of the mice were measured. The gray dashed lines represent the normal serum ALT levels, 40 U/L. (B) Flow cytometric analysis of the levels of activated CTLs in the livers. Intrahepatic lymphocytes were isolated and stained for CD3 (PerCP), CD8 (FITC), and CD69 (PE). The percentages of CD69-positive cells in the CD3- and CD8-double positive lymphocytes were determined by flow cytometry. In addition, the liver expression of (C) IFN-γ, (D) TNF-α, (E) CXCL9, and (F) CXCL10 was analyzed by qRT-PCR. Values are shown as the mean ± SD.

To pursue the mechanisms underlying the different clearance rates of HBeAg in BALB/c, C57BL/6, and FVB/N mice, we examined the severity of liver damage, the presence of activated CTLs, and the status of liver inflammation. Serum ALT levels in the DNA-injected animals were measured in order to determine the severity of liver damage. As shown in Fig. 3A, a huge and transient ALT peak (1500–2000 U/L) was immediately observed one day after DNA injection in all three mouse strains tested, most likely due to the acute liver damage induced by hydrodynamic injection. A second, moderate ALT peak was observed in the BALB/c mice at 2 wpi (∼200 U/L), and in the C57BL/6 mice at 6 wpi (∼150 U/L). In both mouse strains, the ALT levels eventually normalized. However, the ALT levels in the FVB/N mice, once recovering from the acute liver damage, remained slightly elevated relative to the normal range (between 40–100 U/L), a state that was maintained for at least 29 weeks (data not shown). The results indicate that injection of HBV DNA in FVB/N mice induces less liver damage than in BALB/c and C57BL/6 mice.

Effector CD8^+^ CTLs are thought to play an important role in the clearance of HBV infection; they are also implicated in the pathogenesis of liver diseases [29], [30]. Therefore, we examined the levels of activated CTLs in the BALB/c, C57BL/6, and FVB/N mouse strains after HBV DNA injection. Intrahepatic lymphocytes (IHLs) were isolated and analyzed by flow cytometry. As shown in Fig. 3B and Fig. S2, the levels of CD3^+^CD8^+^CD69^+^ activated CTLs were elevated as early as 2 wpi in the BALB/c mice (∼40%), and they peaked at 3 wpi in the C57BL/6 mice (∼25%). IFN-γ enzyme-linked immunospot (ELISPOT) assays demonstrated that the CTLs isolated from the peak levels displayed significant reactivities to the HBs, HBc, or polymerase peptides (Fig. S3 and Methods S1), suggesting that they were HBV-specific. On the other hand, the levels of activated CTLs in the FVB/N mice were not significantly elevated (<5%) throughout the entire course of the experiment. Thus, the levels of activated CTL strongly correlated with the severity of liver damage in BALB/c, C57BL/6, and FVB/N mice.

Next, we examined the inflammatory status of the livers in the DNA-injected animals. The liver expression of inflammatory or pro-inflammatory cytokines, such as IFN-γ and TNF-α, was examined by quantitative reverse transcription PCR (qRT-PCR). In both the BALB/c and C57BL/6 mouse strains, IFN-γ (Fig. 3C) and TNF-α (Fig. 3D) were significantly induced slightly prior to or simultaneously with ALT elevation, whereas the expression of IFN-γ and TNF-α remained low in the FVB/N mice. The small peaks of IFN-γ or TNF-α expression in the FVB/N mice during the first few days after the injection were most likely due to the minimal liver damage caused by hydrodynamic injection, as injection with pGEM4Z vector also demonstrates similar peaks.

**Figure 4:**
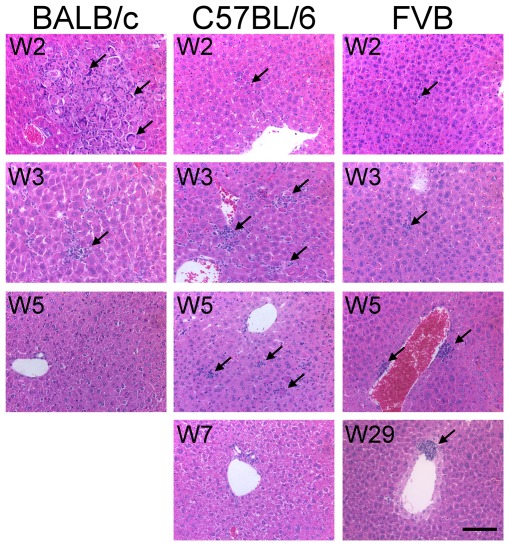
Histological analysis of the livers from different mouse strains injected with HBV DNA. BALB/c, C57BL/6, and FVB/N mice were injected with the pHBV1.3-B6 replicon DNA. The mice were sacrificed at the time points indicated, and their livers were fixed with formalin, embedded in paraffin, and stained with hematoxylin and eosin. The arrows indicate the infiltrating inflammatory cells. The scale bar represents 100 µm. The images are displayed at 200× magnification.

Previous studies by Guidotti *et al.* have shown that the IFN-γ expressed by activated CTLs not only clears HBV non-cytopathically but also induces the expression of chemokines such as CXCL9 and CXCL10, which in turn recruit antigen non-specific mononuclear cells, resulting in liver pathogenesis and viral clearance [29], [30]. Because we observed different levels of IFN-γ and TNF-α in the different mouse strains studied, we compared the expression levels of CXCL9 and CXCL10 in the livers of these mouse strains. As expected, both CXCL9 and CXCL10 were expressed at the highest levels in the BALB/c mice, at the second highest levels in the C57BL/6 mice, and at the lowest levels in the FVB/N mice (Figs. 3E and 3F). The hematoxylin and eosin (H&E) staining results revealed the inflammatory status of the livers, as shown in Fig. 4. The livers of the BALB/c mice exhibited severe inflammation at 2 wpi, which was reduced at 3 wpi and normalized at 5 wpi. Liver inflammation in the C57BL/6 mice was much more moderate relative to that of the BALB/c mice. Intrahepatic inflammatory cells first appeared at 2 wpi, significantly increased at 3–5 wpi, and returned to normal at 7 wpi. Not surprisingly, the livers of the FVB/N mice displayed only slight infiltration of inflammatory cells from 2 wpi to 29 wpi.

Collectively, these data indicate that the injection of HBV DNA into the liver induces different degrees of T-cell activation and liver inflammation in different mouse strains, and the mice can be ordered by the degree of inflammation as follows: BALB/c mice >C57BL/6 mice >FVB/N mice. The degree of liver inflammation strongly correlated with the animal's ability to clear the HBV DNA.

### HBV viral sequences affect HBV persistence in FVB/N mice

**Figure 5:**
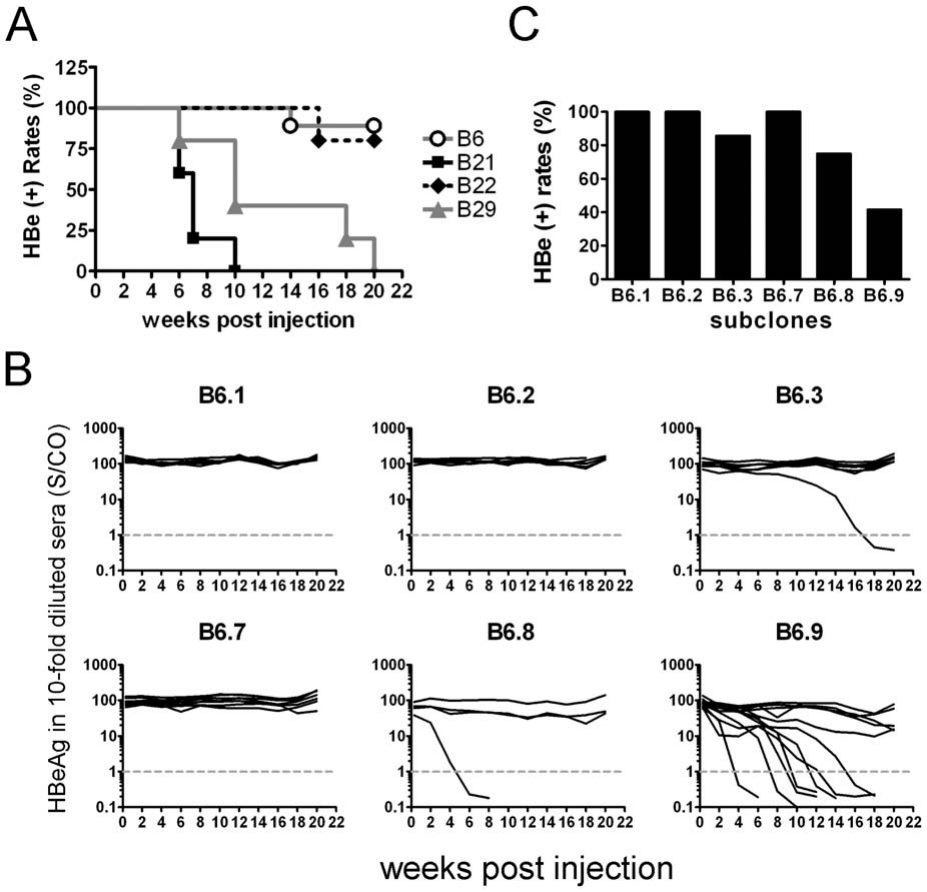
Different persistence rates of different HBV isolates. (A) The replicon DNA used for injection included pHBV1.3-B6, -B21, -B22, and -B29 isolated from different patients. The kinetics of HBeAg clearance over a period of 20 weeks are shown. (B) The replicon DNA of the six sub-clones (B6.1, B6.2, B6.3, B6.7, B6.8, and B6.9) contained in the B6 clone was injected separately into the FVB/N mice. Serum HBeAg was monitored for 20 weeks. The dotted gray lines represent the cut-off value of HBeAg. (C) The HBeAg-positive rate of each sub-clone is shown at 20 weeks post injection.

The low levels of IFN-γ and inflammatory cells in the FVB/N mice may suggest that this mouse model has little or no ability to induce CTL activity in response to HBV replication. However, although clone B6 and another HBV isolate (B22) persistently replicated in the FVB/N mouse livers, other HBV isolates from different patients (e.g., B21 and B29) were cleared within 10–20 weeks (Fig. 5A). The lower persistence rates of clones B21 and B29, however, were not due to the lower replication or gene expression efficiency of these clones relative to that of the B6 and B22 clones (Fig. S4). These data suggested that FVB/N mice were capable of mounting an immune response to clear HBV, although this response remained weaker than that of BALB/c mice. With regard to the B6 clone, we speculate that this clone may possess inherent characteristics that allow it to escape the CTL response in FVB/N mice.

We next investigated the impact of viral factors on HBV persistence. The persistence rates were analyzed among the six sub-clones (B6.1, B6.2, B6.3, B6.7, B6.8, and B6.9) contained in the B6 clone. These sub-clones had <0.5% nucleotide sequence diversity compared to the B6 consensus sequences (data not shown). Interestingly, when injected separately into the FVB/N mice, clone B6.9 demonstrated a lower persistent rate than did the others (Figs. 5B and 5C). Sequence analysis revealed that clone B6.9 had mutations at amino acid 337 of the polymerase (H337Y) and at amino acid 214 of the surface protein (N214S), both mutations that were not present in other sub-clones (Table 1).

**Table 1:** Mutation sites of the amino acid residues in the six sub-clones compared to the B6 consensus sequence.

	Amino acid changes in ORF			
Sub-clone	X	C	S	P
B6.1	identical	identical	L200H	identical
B6.2	G44D, N149T	identical	identical	V302M, V805M
B6.3	identical	identical	E39A	R219S
B6.7	identical	identical	P69L	D126E, V302M, C602G
B6.8	A12T	identical	I68T, T125A	H305R, F307V, C602G
B6.9	G44D	identical	**N214S**	**H337Y**, V805M

**Figure 6:**
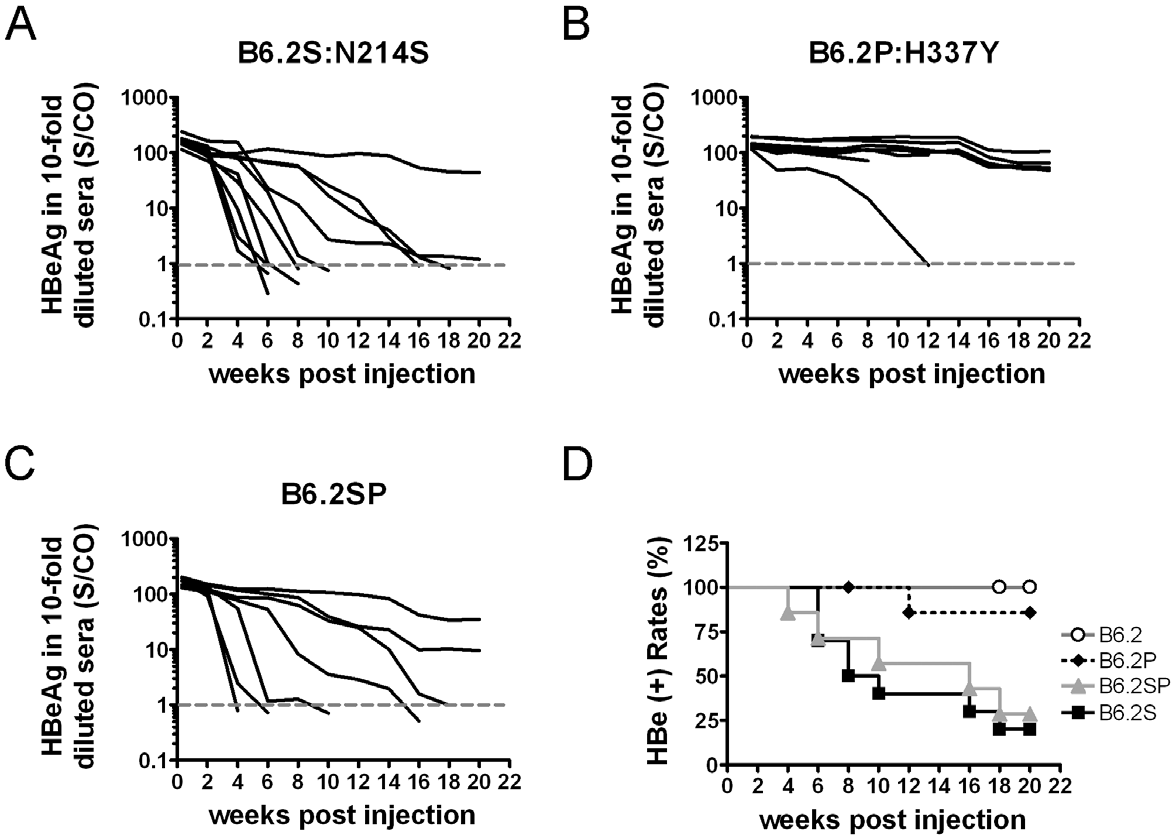
Different persistence rates conferred by a single amino acid change in the HBV surface protein. The mutant replicon DNAs, including (A) pHBV1.3-B6.2S, containing an N214S point mutation in surface protein; (B) pHBV1.3-B6.2P, containing an H337Y point mutation in polymerase; and (C) pHBV1.3-B6.2SP, containing both N214S and H337Y mutations, were injected into FVB/N mice. The levels of HBeAg in 10-fold diluted sera are shown as S/CO, signal-to-control ratio. Each line represents one animal. The dotted gray lines represent the cut-off value of HBeAg. (D) The kinetics of HBeAg clearance in the mice injected with the wild-type sub-clone B6.2 or the three mutants (B6.2S, B6.2P, and B6.2SP) are shown and were compared using the logrank test. The HBeAg-positive rates are B6.2S<B6.2 (**P<0.01), B6.2SP<B6.2 (*P<0.05), and B6.2P *vs.* B6.2, no significance.

Therefore, we investigated whether the variation of these two amino acid residues conferred the susceptibility of clone B6.9 to immune responses. The sequence of clone B6.2, one of the highly persistent sub-clones (Fig. 5B), is the closest to the sequence of clone B6.9. As a result, we performed site-directed mutagenesis on clone B6.2 for persistence analysis. Clone B6.2S contains an N214S mutation in the surface protein region, clone B6.2P contains an H337Y mutation in the polymerase region, and clone B6.2SP has both mutations. When injected separately into the FVB/N mice, clones B6.2S (Fig. 6A) and B6.2SP (Fig. 6C) showed significantly higher clearance rates than did the parental clone (B6.2), whereas clone B6.2P (Fig. 6B) showed no significant difference in clearance compared with the parental strain (Fig. 6D). These results clearly indicate that the Ser-214 residue in the surface protein represents a critical residue responsible for the clearance of clone B6.9 in the FVB/N mice and indicate that a one-residue difference (B6.2S *vs.* B6.2) can lead to significantly different persistence rates.

**Figure 7:**
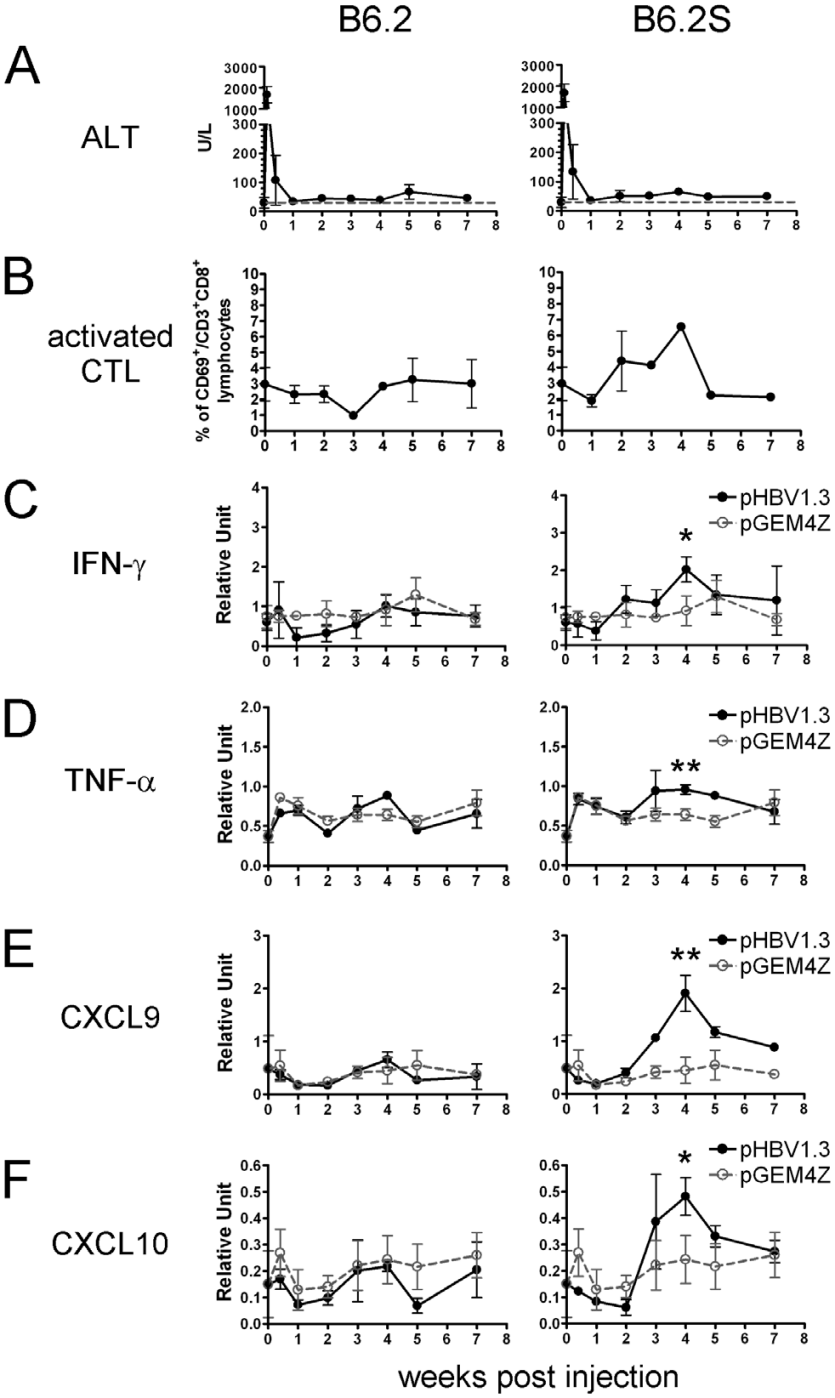
The levels of ALT, activated CTLs, and the liver expression of cytokines and chemokines in FVB/N mice following injection with pHBV1.3-B6.2 or -B6.2S DNA. Mice were injected with pHBV1.3-B6.2 or -B6.2S DNA (10 µg/mouse). Three animals were sacrificed at the time points indicated. (A) Serum ALT levels of the mice were measured. The gray dashed lines represent the normal serum ALT levels, 40 U/L. (B) Flow cytometric analysis of activated CTLs in the livers. Staining of activated CTLs was performed as described in the legend to Fig. 3B. The liver expression of (C) IFN-γ, (D) TNF-α, (E) CXCL9, and (F) CXCL10 was analyzed by qRT-PCR. Values are shown as the mean ± SD of three mice. *P<0.05; **P<0.01.

**Figure 8:**
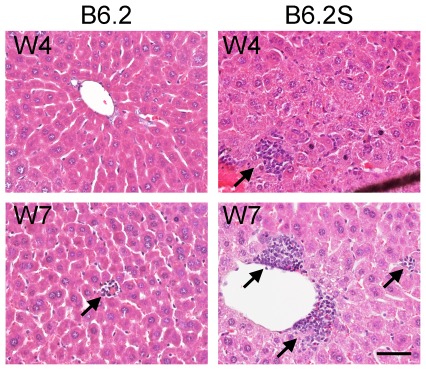
Histological analysis of the livers of FVB/N mice injected with pHBV1.3-B6.2 or -B6.2S replicon DNA. FVB/N mice were injected with pHBV1.3-B6.2 or -B6.2S replicon DNA. The mice were sacrificed at the indicated time points, and their livers were fixed with formalin, embedded in paraffin, and stained with hematoxylin and eosin. The arrows indicate the infiltrating inflammatory cells. The scale bar represents 50 µm. The images are displayed at 400× magnification.

Next, we examined whether the clearance of clone B6.2S was also associated with the induction of inflammation. Although serum ALT levels were not significantly elevated in the FVB/N mice injected with pHBV1.3-B6.2 or -B6.2S (Fig. 7A), the levels of activated CTLs were increased in the mice injected with B6.2S (∼6%) compared with those injected with B6.2 (Fig. 7B). This CTL increase was accompanied with slight, but significant, elevations in the levels of intrahepatic IFN-γ, TNF-α, CXCL9, and CXCL10 (Figs. 7C–7F). The H&E staining results showed that the livers of the FVB/N mice injected with clone B6.2 exhibited little to no infiltration of inflammatory cells, whereas the FVB/N mice injected with clone B6.2S demonstrated more infiltration of inflammatory cells, especially at 7 wpi (Fig. 8).

The epitope sequence motifs restricted to H-2^q^ have not been reported. Thus, it is not clear whether the single N214S mutation may generate a potential CTL epitope leading to the clearance of clone B6.2S in the FVB/N mice. To test this possibility, we performed ELISPOT assays using the IHLs isolated from the animals injected with B6.2S. The IHLs were pulsed with two 15-mer peptides that had the wild-type or the N214S mutated residue located in the center of the 10-amino acid overlapped region (Fig. S5A). It is anticipated that these two peptides, once processed, be able to generate octapeptide epitopes that have the mutated residue possibly located at position 1 to 8. However, the results of the ELISPOT assays did not detect any peptide-specific CTL response either with the wild-type or with the N214S peptide (Fig. S5B). We speculated that the CTL frequency in the FVB/N mice might be too low to be detected as the CTL activation and inflammatory responses in the FVB/N mice injected with the B6.2S DNA were much lower than those observed in the BALB/c mice injected with pHBV1.3-B6 DNA (Fig. 3 *vs.* Fig. 7). Therefore, we used an alternative approach to determine whether this mutated sequence could serve as a potential epitope to induce CTLs. FVB/N mice were intramuscularly immunized with a plasmid DNA expressing either the wild-type or the mutated HBsAg containing the N214S residue and were subsequently boosted every two weeks for three times (Methods S1). An ELISPOT assay using the aforementioned peptides was performed on the splenocytes one week after the final boost. However, even with such vigorous immunization procedures, the peptide-specific T-cell responses were still not detected in the FVB/N mice (Fig. S5C), whereas HBsAg-specific T-cell responses were easily detected in the C57BL/6 mice with just two immunizations (Fig. S5D). These results may suggest that the N214S mutated sequence is not the T-cell epitope. However, this possibility has not been formerly excluded.

Collectively, these results reinforced the association of HBV clearance with the activation of CTLs, IFN-γ expression, and the subsequent induction of inflammation. Moreover, they indicate that a difference in a single or a few amino acids in HBV can lead to different immune responses and differences in the ultimate fate of the virus.

## Discussion

In this study, we have employed hydrodynamic injection to introduce HBV replicon DNA into mouse livers. Whereas the replicon DNA was rapidly cleared from the livers of the BALB/c and C57BL/6 mice in four and eight weeks, respectively, it persistently replicated and induced low levels of ALT elevation in the livers of the FVB/N mice for up to 50 weeks. It is noteworthy that FVB/N mice possess competent immune responses as evidenced by the complete clearance of some HBV clones (Fig. 5A). However, an estimated 50% of the HBV clones from clinical isolates were able to replicate persistently in the FVB/N mice (data not shown), indicating that persistent replication is not an isolated event for the HBV1.3-B6 clone. Although 100% of the isolates were cleared in BALB/c and C57BL/6 mice, the factors that allow persistent HBV replication in FVB/N mice are peculiar and interesting and were explored in this study.

In humans, there is a clear difference in the adaptive immunity between patients with persistent, chronic HBV infections and patients who resolve the infection. The development of strong HBV-specific cellular immunity with Th1-type cytokine production is usually associated with the resolution of the HBV infection, whereas a weaker or undetectable cellular immune response that is unable to control HBV replication is present in patients with chronic HBV infection [13], [31]. Moreover, through the use of transgenic mice and adoptive transfer techniques, Guidotti and Chisari have demonstrated that both CTLs and antigen-nonspecific inflammatory cells are important for viral clearance [29], [30]. In line with these reports, our results demonstrated that the HBV clearance strongly correlates with the presence of intrahepatic CTLs and the severity of the liver inflammation during viral infection. Using genetic approaches, we further demonstrated that viral sequences also played influential roles in determining the HBV persistence. A single amino acid change of Asn-214 to Ser-214 in the HBsAg rendered an otherwise persistent HBV clone clearable in FVB/N mice. Collectively, the HBV persistence in the nontransgenic FVB/N mouse model is attributable to two essential factors: the genetic background of the host and the appropriate viral sequences.

FVB/N mice have an H-2^q^ MHC haplotype and an Hc^0^ allele. Macrophages from the mouse strains carrying the Hc^0^ allele do not secrete complement 5 [32], an important factor in inflammation and in innate immune responses [33]–[35], whereas MHC molecules are involved in antigen presentation. Therefore, we speculate that the H-2^q^ allele may represent a special MHC haplotype in mice, akin to the HLA-DP in humans [17], that induces weaker T-cell responses against HBV. However, H-2^q^ *per se* may not completely explain the high HBV persistence rates in this mouse strain. We have other experimental results showing that DBA/1 mice, a strain that also carries the H-2^q^ allele, were actually capable of clearing the HBV DNA with 78% efficiency, albeit over a long period (4–16 weeks, Fig. S6). Therefore, we hypothesize that the additional defects in the secretion of C5 complement by the macrophages of FVB/N mice, which leads to lower levels of inflammatory responses, may constitute another factor contributing to the high HBV persistence rates. As such, even if some HBV clones could be cleared in FVB/N mice (e.g., B21, B29, and B6.2S), the lower inflammatory response still resulted in the delayed clearance of the HBV DNA in FVB/N mice compared to that in the BALB/c and C57BL/6 mice (Fig. 5A *vs.* Fig. 1D).

Recently, many different mouse models have been established to investigate the pathogenesis and persistence of HBV infection. The HBV transgenic mice, though invaluable in understanding the pathogenesis induced by the adoptively transferred CTLs [15], are inherently tolerant to the transgene products and thus are unsuitable to address the question of HBV persistence. Many researchers have established HBV replication models in immunocompetent nontransgenic mice by employing hydrodynamic injection [36] or adenoviral vector delivery [37], [38], but in most cases only acute hepatitis can be demonstrated, and the HBV infection is eventually cleared. Persistent HBV replication in a mouse model has been demonstrated only under special conditions in which the HBV DNA was cloned in an adeno-associated viral (AAV) vector and then delivered to mouse livers either via hydrodynamic injection [39] or via AAV infection [40]. The mechanism for this persistence is partly dependent on the AAV vector, which has been shown to be poorly detected by host innate immunity and favors long-term persistence in the cells [41], [42]. On the other hand, the HBV persistence in our FVB/N model is most likely due to host effects rather than to vector effects. HBV infection in immunocompetent adults usually results in a self-limited, transient liver disease in more than 95% of patients. FVB/N mice are akin to the remaining 5% of patients who may have unique HLAs and induce weaker adaptive and/or innate immune responses against HBV, thus having a higher tendency of developing a persistent infection. In concert with this speculation, genetic variants in MHC genes [11], [12], the TNF-α gene [8]–[10], and the IFN-γ gene [7] have been reported to be associated with chronic hepatitis B infection in humans.

Our mutagenesis data reveal that FVB/N mice are unable to clear clone B6.2 but can clear clone B6.2S (Fig. 6). Clone B6.2S differs from clone B6.2 by just one amino acid. We originally speculated that the mutation site might have generated a novel CTL epitope recognizable by FVB/N mice, but our attempts at demonstrating the presence of such an epitope in this region have failed. This failure either implies that the mutation does not generate an epitope or may be due to the low CTL frequency induced in FVB/N mice. If the former case is true, then we speculate that the mutation may alternatively induce a conformational change of the protein and result in a different pattern of antigen processing, which may create a CTL epitope elsewhere. However, due to the lack of epitope information for H-2^q^ and a suitable cell line carrying this haplotype allowing for a CTL assay, the speculation that Asn-214 contributes to T-cell tolerance is unproven and remains to be elucidated.

In summary, the unique H-2^q^ allele of FVB/N mice may activate weaker CTL responses after HBV DNA injection, and the Hc^0^ allele concurrently induces lower inflammatory responses; either or both factors may provide FVB/N mice with the capability of establishing persistent HBV replication more easily than would other mouse strains. With the ease of hydrodynamic injection and the convenience of genetic engineering techniques, these mouse strains, each with a different susceptibility to persistent HBV replication, offer a great opportunity to investigate the host and viral factors associated with HBV persistence *in vivo*.

## Materials and Methods

### Ethics statement

Serum samples were collected from the outpatients at National Taiwan University Hospital with written informed consent. The study was approved by National Taiwan University Hospital Research Ethics Committee (Permit Number 200809073R). The animal study was carried out in strict accordance with the recommendations in the “Guide for the Care and Use of Laboratory Animals” of the Council of Agriculture, Taiwan, and that of the National Institutes of Health, USA. The protocol has been reviewed and approved by the Institutional Animal Care and Use Committee of National Yang-Ming University (Approval No. 971017). All surgery was performed under ketamine and xylazine anesthesia, and all efforts were made to minimize suffering.

### Construction of HBV replicons

HBV replicons containing a replication-competent 1.3-fold overlength HBV genome were used for hydrodynamic injection. To construct the replicons, the full-length HBV genomes were amplified from clinical isolates of genotype B HBV that were obtained from serum samples of outpatients at the National Taiwan University Hospital. HBV DNA was extracted from the serum using QIAamp® DNA Mini Kit (QIAGEN, Hilden, Germany) following the manufacturer's instructions and was then amplified by polymerase chain reaction (PCR) using the primer pair P1–P2 [43]. The sequences of the primers are shown in Table S1. The PCR products were cloned in the yT&A vector (Yeastern Biotech, Taipei, Taiwan). Linear HBV monomers were then released from the vector by cleavage with 1.5 U of *Sap*I (New England Biolabs, MA) per µg of DNA for at least 12 h and self-ligated with T4 DNA ligase. The self-ligated DNAs were used as the templates for the next PCR. Two sets of primer pairs, Primer-A1 and -A2 and Primer-B1 and -B2 (Table S1), were used to amplify fragments A and B, respectively. Fragment A was then digested with *Hind*III and *Xba*I, and fragment B was digested with *Xba*I and *Bgl*II. The fragments were subsequently cloned into the *Hind*III- and *Bam*HI-digested pGEM4Z vector (Promega, Madison, WI) via a tri-molecular ligation. The resulting pGEM4Z/HBV1.3 replicons contain a 1.3-fold overlength HBV genome spanning from nucleotide (nt) 971 to nt 3215/1 and nt 1 to nt 1984 (Fig. S1). To represent the quasispecies nature, the pHBV1.3-B6 clone DNA was produced by mixing six sub-clone DNAs (B6.1, B6.2, B6.3, B6.7, B6.8, and B6.9) at equal ratios, which were derived from six isolates from the same patient.

Site-directed mutagenesis was performed to generate the pHBV1.3-B6.2 mutant clones using the previously described method [44]. The primers used to generate clone B6.2S and clone B6.2P are Primer-N214S and Primer-H337Y, respectively (Table S1). For pHBV1.3-B6SP, the site-directed mutagenesis was performed twice serially to obtain the double mutant clone.

### Animal experiments

BALB/c, C57BL/6, and FVB mice (male, 6–8 weeks of age) obtained from the National Laboratory Animal Center, Taiwan were housed in a specific pathogen-free room. Ten micrograms of the HBV plasmid DNA in a volume of PBS equivalent to 8% of the mouse body weight was introduced by tail vein injection in 6–8 s. Serum samples were collected at the time points indicated after DNA injection and assayed for HBeAg and ALT.

### Detection of HBeAg and ALT in the serum

Serum levels of HBeAg in the injected mice were determined using the AXSYM system kit (Abbott GmbH Diagnostica, Wiesbaden, Germany). Serum ALT activity was measured by an automated clinical chemistry analyzer TBA-200FR (Toshiba, Tokyo, Japan) using the ALT/GPT reagent (Denka Seiken, Tokyo, Japan).

### Southern blot analysis of HBV DNA in the livers

Twenty micrograms of liver genomic DNA were digested with *Hind*III and separated on a 1% agarose gel. The gel was blotted onto a positively charged nylon membrane (Roche Diagnostics GmbH, Mannheim, Germany), which was then hybridized with a digoxigenin-labeled probe encompassing the HBx coding region (nt 1372–1833), and the signals were detected by exposure to X-ray films.

### Immunohistochemistry

Liver tissues were collected from the mice at the indicated time points, snap-frozen, and sectioned into 5-µm cryosections. Liver sections were stained with rabbit anti-HBcAg (1∶700 dilution, DAKO, Carpinteria, CA) or rabbit anti-HBsAg (ad+ay) (1∶500 dilution, Biodesign, Saco, ME) antibody and then developed using the Envision System-HRP, DAB (DAKO) following the manufacturer's instructions. The liver sections were counterstained with hematoxylin.

### Isolation of intrahepatic lymphocytes

IHLs were isolated following the method described previously [45], with some modifications. Briefly, the livers were perfused with 5 ml of liver digestion medium containing 100 µg/ml collagenase IV (Sigma-Aldrich, St. Louis, MO) and 10 µg/ml DNase I (Roche Diagnostics GmbH, Mannheim, Germany) in HBSS. The livers were then cut into small pieces in 5 ml of the digestion medium and then incubated for 40 min at 37°C. The small liver pieces were further homogenized and forced through a metal mesh. The liver slurry was washed and centrifuged at 50×*g* for 5 min at 4°C. The supernatant was collected and centrifuged at 300×*g* for 10 min at 4°C. The cell pellet was resuspended with ice-cold serum-free RPMI-1640 to a final volume of 4 ml, and 4 ml of 80% (v/v) Percoll (GE Healthcare Bio-Sciences AB, Uppsala, Sweden) in RPMI-1640 was added and mixed well. The mixture was layered on the top of 2 ml of 60% (v/v) Percoll in HBSS and centrifuged at 1,200×g for 20 min at room temperature (RT). The IHLs were harvested from the interface, washed, and used for flow cytometric analysis.

### Flow cytometric analysis

IHLs were stained with the following monoclonal antibodies: peridinin chlorophyll protein (PerCP)-conjugated anti-mouse CD3e, fluorescein isothiocyanate (FITC)-conjugated anti-mouse CD8a, and R-Phycoerythrin (R-PE)-conjugated anti-mouse CD69 (all from BD Biosciences, Mountain View, CA). Flow cytometry data were acquired using a FACSCanto apparatus with FACSDiva software (BD Biosciences) and were analyzed using FlowJo software (Treestar, Inc., San Carlos, CA).

### qRT-PCR analysis of cytokine and chemokine gene expression

Liver RNA was extracted using TRIzol® reagent (Invitrogen, Carlsbad, CA), and 1 µg of RNA was subjected to reverse transcription. One twentieth of the cDNA product was then incubated with specific primers (Table S1) using Fast SYBR Green Master Mix (Applied Biosystems, Carlsbad, CA). The reaction mixture was first denatured at 95°C for 20 sec, and 40 cycles of PCR were performed using the following settings: 95°C for 3 sec and 60°C for 30 sec. Each reaction was performed in triplicate. The expression level of each gene was normalized to beta-actin.

### Statistical analysis

The HBeAg clearance rates in different groups were compared using the logrank test. For statistical analysis of two variables, two-tailed Student's *t*-test was used (GraphPad Prism 4 software, San Diego, CA). A *P*-value<0.05 was considered statistically significant.

## Supporting Information

Figure S1 **The pGEM4Z/HBV1.3 replicon plasmid.** The pGEM4Z/HBV1.3 vector contains the HBV fragment spanning from nt 971 to nt 3,215 and from nt 1 to nt 1,984, which contains a 1.3-fold overlength HBV genome. The restriction enzyme sites used for construction of pGEM4Z/HBV1.3 are shown.
(TIF)

Figure S2 **Flow cytometric analysis of the levels of activated CTLs in the livers of different mouse strains after DNA injection.** Mice were injected with pHBV1.3-B6 DNA (10 µg/mouse), and three animals were sacrificed at the indicated time points after DNA injection. Intrahepatic lymphocytes were isolated and stained for CD3 (PerCP), CD8 (FITC), and CD69 (PE). Representative histograms show the CD69 expression by CD3^+^CD8^+^ cells within a forward and side scatter gate appropriate for lymphocytes (solid lines). Lymphocytes were stained for CD3 (PerCP) and CD8 (FITC) as fluorescence-minus-one controls (shaded plots).
(TIF)

Figure S3 **HBV specific CTL responses detected by IFN-γ ELISPOT assay.** The results of IFN-γ ELISPOT assays for BALB/c (A) or C57BL/6 (B) mice are shown. Mice were injected with pGEM4Z vector or pHBV1.3-B6 DNA (10 µg/mouse), and three animals were sacrificed at 2 wpi (BALB/c mice) or 3 wpi (C57BL/6 mice), respectively. The isolated IHLs and splenocytes of BALB/c mice were stimulated with a peptide pool containing 5 µg/ml of each of the following: P140 (Pol_140–148_), C131 (HBcAg_131–139_), and S28 (HBsAg_28–39_), whereas those of C57BL/6 mice were stimulated with 5 µg/ml of S190 (HBsAg_190–197_, H-2K^b^-restricted). After 18–20 h of peptide stimulation, the frequencies of IFN-γ-secreting cells were determined and measured as the number of spot-forming cells (SFC) per 5×10^5^ cells. Asterisks mean significant difference between the HBV DNA- and the vector-injected animals. Results are shown as the mean ± SD. **P<0.01; *** *P*<0.001.
(TIF)

Figure S4 **The replication and gene expression efficiencies of different HBV isolates.** One microgram of pHBV1.3 DNA was transfected into 7×10^5^ HuH-7 cells. Sixteen hours later, the transfected cells were subcultured for RNA, DNA, protein, and luciferase activity analyses. (A) Northern blot analysis shows HBV gene expression efficiency. Two days after HBV DNA transfection, total RNA was extracted and analyzed by Northern blot with an HBV-specific probe and a GAPDH probe. The sizes corresponding to the HBV transcripts are indicated to the right. (B) Southern blot analysis shows viral DNA replication efficiency. Total genomic DNA was harvested three days after HBV DNA transfection, digested with *Hind*III, and then analyzed by Southern blot with an HBV-specific probe. The HBV replicative intermediates are indicated to the right. (C) Western blot analysis shows viral antigens expression. Total proteins were extracted four days after HBV DNA transfecton and subjected to western blot analysis. The HBV core protein, S proteins, and α-tubulin are indicated to the right. (D, E) ELISA determines the HBsAg and HBeAg levels, respectively. The supernatants were collected on the 3^rd^ day after HBV DNA transfection, and subjected to ELISA analysis. (F) Renilla luciferase activity assay shows the transfection efficiency. Protein lysates were collected on the 4^th^ day after HBV DNA transfection and Renilla luciferase activities were analyzed.
(TIF)

Figure S5 **No peptide-specific T cell response in FVB mice.** (A) The sequences of the synthetic 15-mer peptides. The sequences covering the Asn-214 region are shown at the top of the figure. The four peptides covering the mutated site are depicted by the lines, namely, LS205-N (L-HBsAg_205–219_-N), LS210-N (L-HBsAg_210–224_-N), LS205-S (L-HBsAg_205–219_-S), and LS210-S (L-HBsAg_210–224_-N), where N and S represent Asn or Ser at the 214^th^ residue, respectively. (B) No T cell response specific to the mutated region was observed in the FVB mice injected with HBV DNA. FVB mice were hydrodynamically injected with pGEM4Z vector or pHBV1.3-B6 DNA (10 µg/mouse), and three animals were sacrificed at 4 wpi. The IHLs were isolated and stimulated with 10 µg/ml of LS205-N, LS210-N, LS205-S, or LS210-S. After 18–20 h of peptide stimulation, the frequencies of IFN-γ-secreting cells were determined and measured as the number of spot-forming cells (SFC) per 5×10^5^ cells. (C) No T cell response specific to the mutated region was observed in the FVB mice intramuscularly immunized with HBsAg-expressing DNA. FVB mice were immunized with control vector or the plasmid DNA encoding HBsAg-B6.2 or HBsAg-B6.2S (100 µg/mouse). The animals were boosted with the same dose of DNA every two weeks for three times. One week after the final boost, HBsAg-specific T-cell response was examined by IFN-γ ELISPOT assays using the aforementioned peptides. The splenocytes isolated from the immunized mice (n = 3) were stimulated with 10 µg/ml of the 15-mer synthetic peptides as described above. After 18–20 h of incubation, the frequencies of IFN-γ-secreting cells were counted. (D) A significant HBsAg-specific T cell response was observed in the C57BL/6 mice. The C57BL/6 mice immunized with a plasmid DNA encoding the HBsAg-B6.2S served as the positive control for DNA immunization. One week after the second immunization, an HBsAg-specific IFN-γ ELISPOT assay was performed on the splenocytes isolated from the immunized mice (n = 3), which were stimulated with 10 µg/ml of HBsAg_190–197_. After 18–20 h of peptide stimulation, the spots of IFN-γ-secreting cells were counted. The results are shown as the mean ± SD. Asterisks mean significant difference between the peptide stimulated- and medium control- groups. *** *P*<0.001.
(TIF)

Figure S6 **Long-term follow up of serum HBeAg in DBA/1 mice injected with pHBV1.3-B6 DNA.** Ten micrograms of pHBV1.3-B6 DNA were injected into DBA/1 mice. (A) Serum HBeAg was monitored regularly for up to 16 weeks. Each line represents one animal. (B) The HBeAg clearance curves in FVB mice and DBA/1 mice are shown. DBA/1 *vs.* FVB, *** *P*<0.001, logrank test.
(TIF)

Table S1 **The sequences of primers used for construction of HBV replicons, site-directed mutagenesis, and real-time PCR analysis.**
(DOC)

Methods S1 IFN-γ ELISPOT assay. DNA immunization.
(DOC)
