# Supplementary figures and images for: Persistent Hepatitis B Viral Replication in a FVB/N Mouse Model: Impact of Host and Viral Factors
Source: PLoS One. 2012 May 16;7(5):e36984. doi: 10.1371/journal.pone.0036984 (PMC3353969; doi:10.1371/journal.pone.0036984)

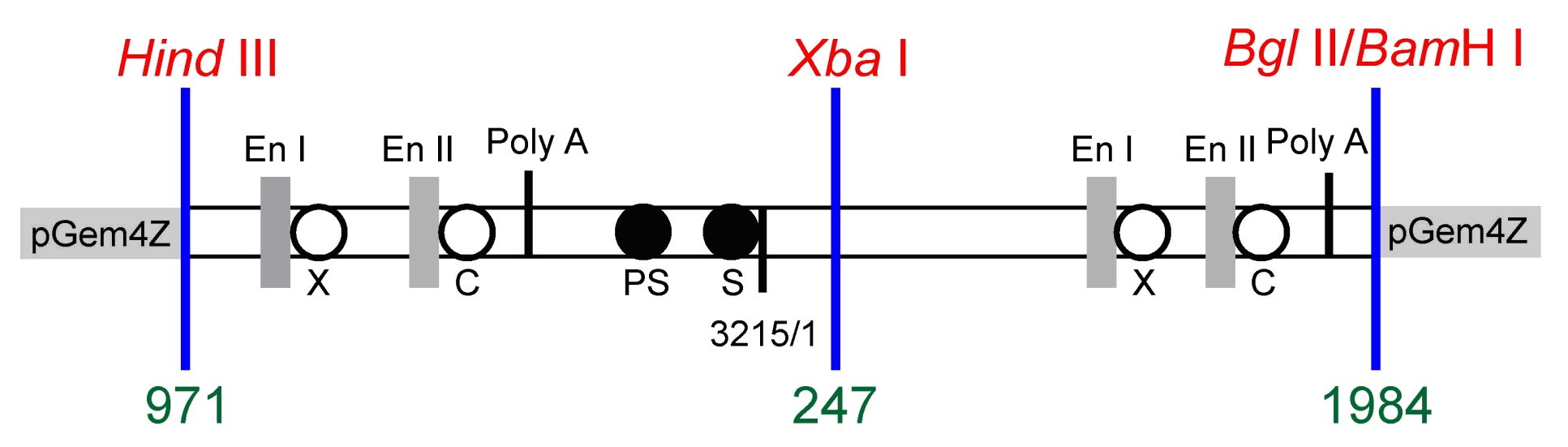

Supplement: Figure S1 — The pGEM4Z/HBV1.3 replicon plasmid. The pGEM4Z/HBV1.3 vector contains the HBV fragment spanning from nt 971 to nt 3,215 and from nt 1 to nt 1,984, which contains a 1.3-fold overlength HBV genome. The restriction enzyme sites used for construction of pGEM4Z/HBV1.3 are shown. (TIF) [file pone.0036984.s001.tif]

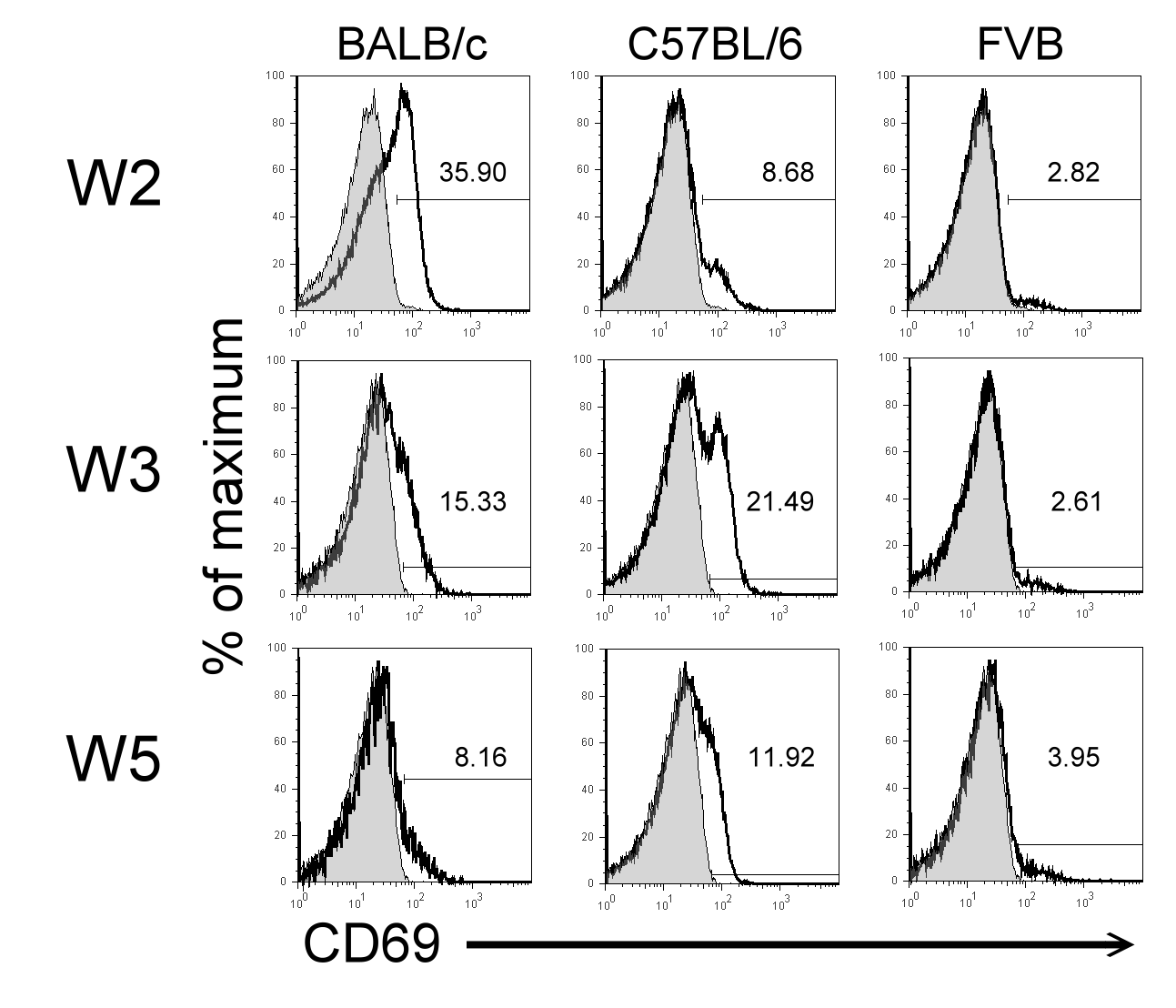

Supplement: Figure S2 — Flow cytometric analysis of the levels of activated CTLs in the livers of different mouse strains after DNA injection. Mice were injected with pHBV1.3-B6 DNA (10 µg/mouse), and three animals were sacrificed at the indicated time points after DNA injection. Intrahepatic lymphocytes were isolated and stained for CD3 (PerCP), CD8 (FITC), and CD69 (PE). Representative histograms show the CD69 expression by CD3+CD8+ cells within a forward and side scatter gate appropriate for lymphocytes (solid lines). Lymphocytes were stained for CD3 (PerCP) and CD8 (FITC) as fluorescence-minus-one controls (shaded plots). (TIF) [file pone.0036984.s002.tif]

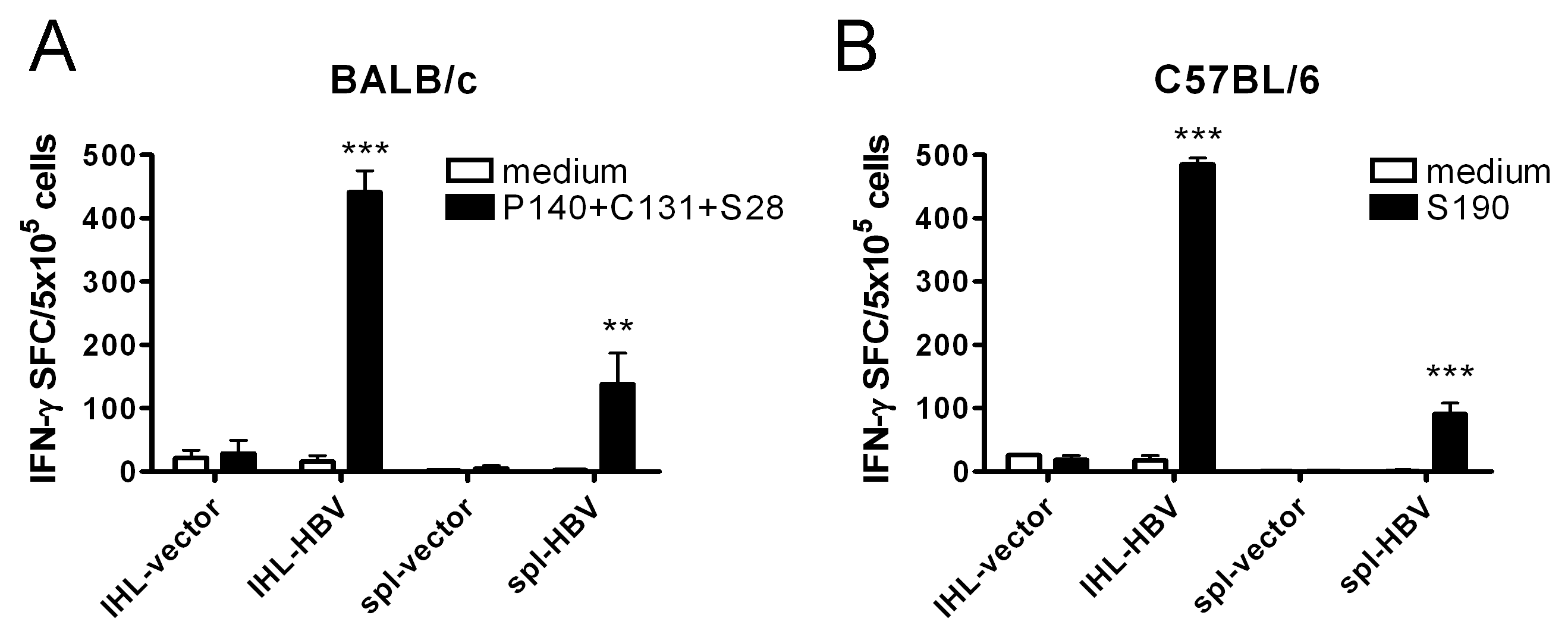

Supplement: Figure S3 — HBV specific CTL responses detected by IFN-γ ELISPOT assay. The results of IFN-γ ELISPOT assays for BALB/c (A) or C57BL/6 (B) mice are shown. Mice were injected with pGEM4Z vector or pHBV1.3-B6 DNA (10 µg/mouse), and three animals were sacrificed at 2 wpi (BALB/c mice) or 3 wpi (C57BL/6 mice), respectively. The isolated IHLs and splenocytes of BALB/c mice were stimulated with a peptide pool containing 5 µg/ml of each of the following: P140 (Pol140–148), C131 (HBcAg131–139), and S28 (HBsAg28–39), whereas those of C57BL/6 mice were stimulated with 5 µg/ml of S190 (HBsAg190–197, H-2Kb-restricted). After 18–20 h of peptide stimulation, the frequencies of IFN-γ-secreting cells were determined and measured as the number of spot-forming cells (SFC) per 5×10^5^ cells. Asterisks mean significant difference between the HBV DNA- and the vector-injected animals. Results are shown as the mean ± SD. **P<0.01; *** P<0.001. (TIF) [file pone.0036984.s003.tif]

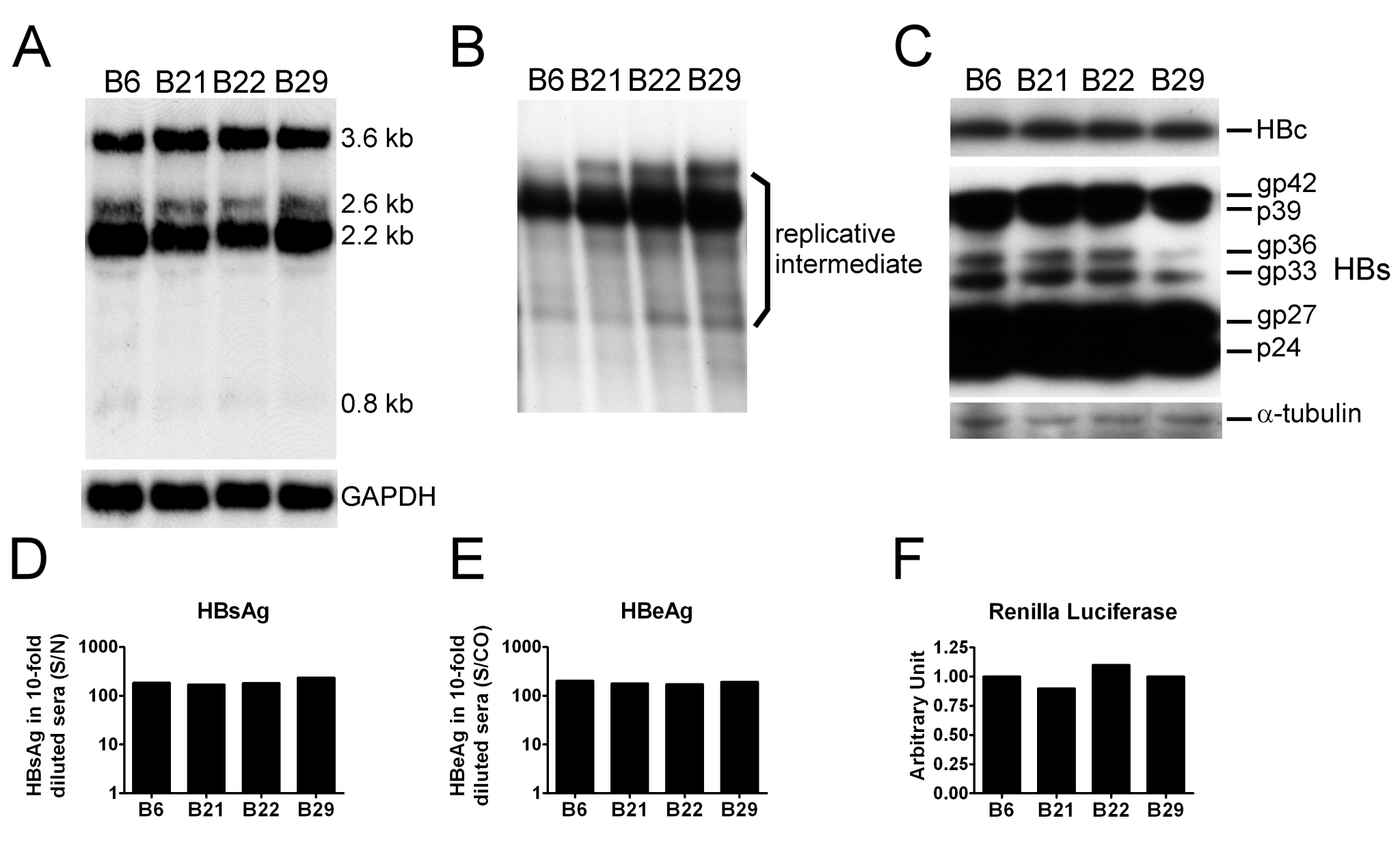

Supplement: Figure S4 — The replication and gene expression efficiencies of different HBV isolates. One microgram of pHBV1.3 DNA was transfected into 7×10^5^ HuH-7 cells. Sixteen hours later, the transfected cells were subcultured for RNA, DNA, protein, and luciferase activity analyses. (A) Northern blot analysis shows HBV gene expression efficiency. Two days after HBV DNA transfection, total RNA was extracted and analyzed by Northern blot with an HBV-specific probe and a GAPDH probe. The sizes corresponding to the HBV transcripts are indicated to the right. (B) Southern blot analysis shows viral DNA replication efficiency. Total genomic DNA was harvested three days after HBV DNA transfection, digested with HindIII, and then analyzed by Southern blot with an HBV-specific probe. The HBV replicative intermediates are indicated to the right. (C) Western blot analysis shows viral antigens expression. Total proteins were extracted four days after HBV DNA transfecton and subjected to western blot analysis. The HBV core protein, S proteins, and α-tubulin are indicated to the right. (D, E) ELISA determines the HBsAg and HBeAg levels, respectively. The supernatants were collected on the 3rd day after HBV DNA transfection, and subjected to ELISA analysis. (F) Renilla luciferase activity assay shows the transfection efficiency. Protein lysates were collected on the 4th day after HBV DNA transfection and Renilla luciferase activities were analyzed. (TIF) [file pone.0036984.s004.tif]

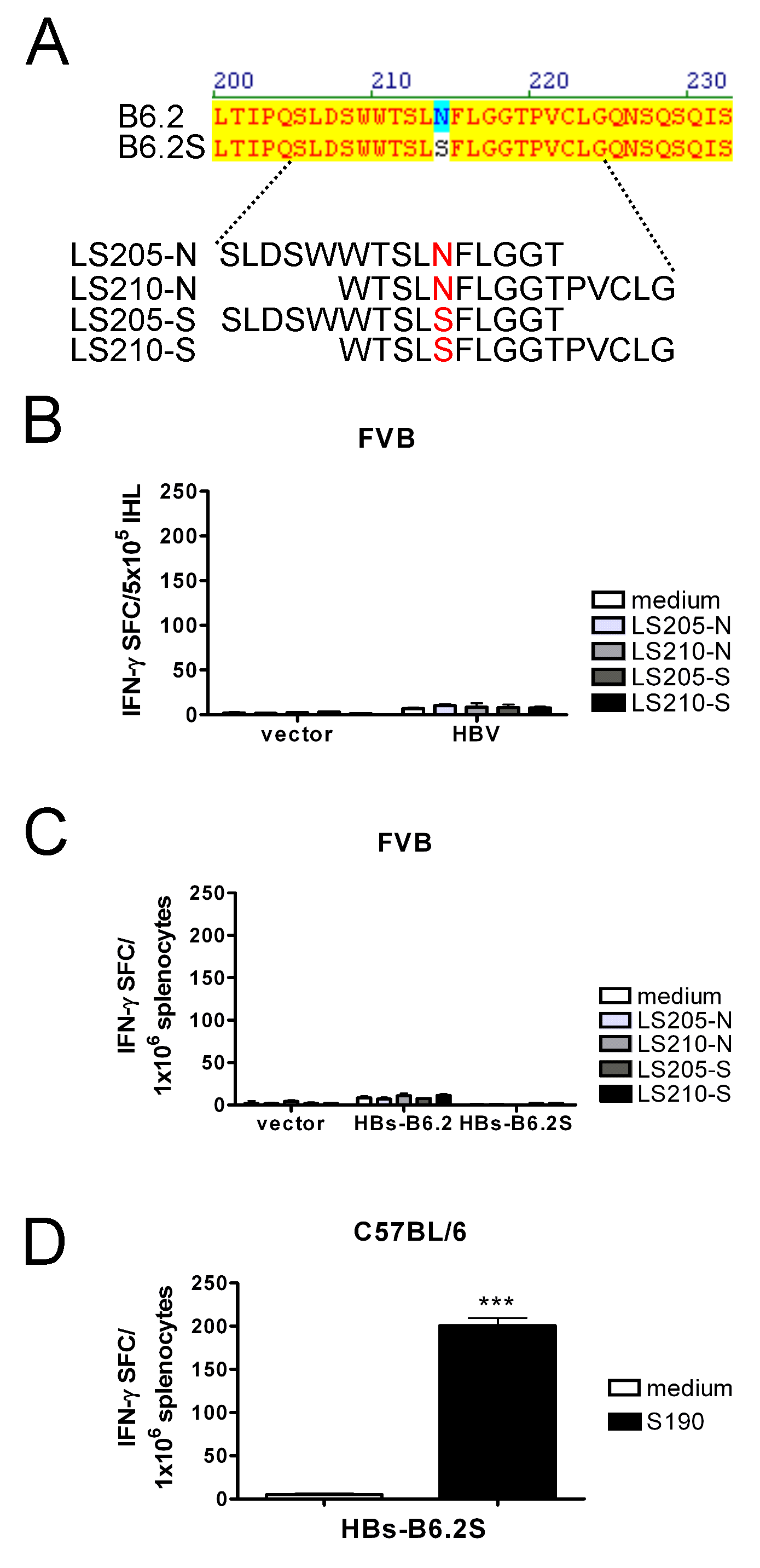

Supplement: Figure S5 — No peptide-specific T cell response in FVB mice. (A) The sequences of the synthetic 15-mer peptides. The sequences covering the Asn-214 region are shown at the top of the figure. The four peptides covering the mutated site are depicted by the lines, namely, LS205-N (L-HBsAg205–219-N), LS210-N (L-HBsAg210–224-N), LS205-S (L-HBsAg205–219-S), and LS210-S (L-HBsAg210–224-N), where N and S represent Asn or Ser at the 214th residue, respectively. (B) No T cell response specific to the mutated region was observed in the FVB mice injected with HBV DNA. FVB mice were hydrodynamically injected with pGEM4Z vector or pHBV1.3-B6 DNA (10 µg/mouse), and three animals were sacrificed at 4 wpi. The IHLs were isolated and stimulated with 10 µg/ml of LS205-N, LS210-N, LS205-S, or LS210-S. After 18–20 h of peptide stimulation, the frequencies of IFN-γ-secreting cells were determined and measured as the number of spot-forming cells (SFC) per 5×10^5^ cells. (C) No T cell response specific to the mutated region was observed in the FVB mice intramuscularly immunized with HBsAg-expressing DNA. FVB mice were immunized with control vector or the plasmid DNA encoding HBsAg-B6.2 or HBsAg-B6.2S (100 µg/mouse). The animals were boosted with the same dose of DNA every two weeks for three times. One week after the final boost, HBsAg-specific T-cell response was examined by IFN-γ ELISPOT assays using the aforementioned peptides. The splenocytes isolated from the immunized mice (n = 3) were stimulated with 10 µg/ml of the 15-mer synthetic peptides as described above. After 18–20 h of incubation, the frequencies of IFN-γ-secreting cells were counted. (D) A significant HBsAg-specific T cell response was observed in the C57BL/6 mice. The C57BL/6 mice immunized with a plasmid DNA encoding the HBsAg-B6.2S served as the positive control for DNA immunization. One week after the second immunization, an HBsAg-specific IFN-γ ELISPOT assay was performed on the splenocytes isolated from the immunized mice (n = 3), [file pone.0036984.s005.tif]

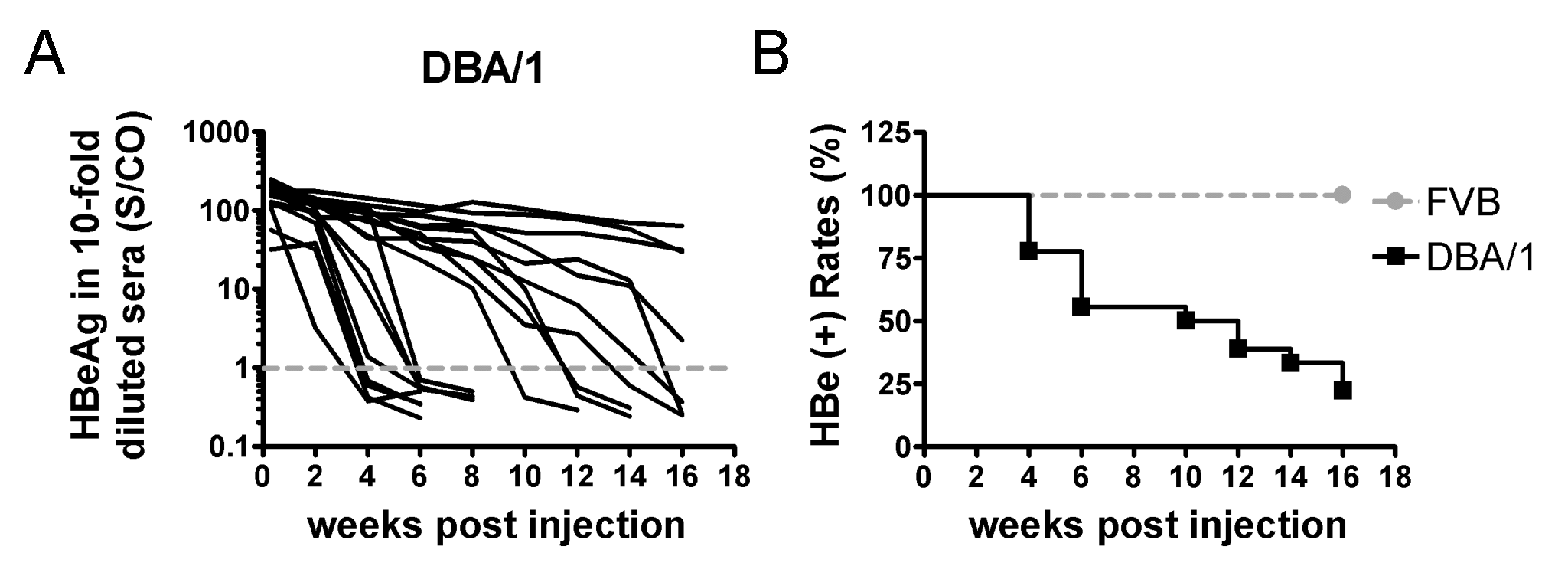

Supplement: Figure S6 — Long-term follow up of serum HBeAg in DBA/1 mice injected with pHBV1.3-B6 DNA. Ten micrograms of pHBV1.3-B6 DNA were injected into DBA/1 mice. (A) Serum HBeAg was monitored regularly for up to 16 weeks. Each line represents one animal. (B) The HBeAg clearance curves in FVB mice and DBA/1 mice are shown. DBA/1 vs. FVB, *** P<0.001, logrank test. (TIF) [file pone.0036984.s006.tif]
