# Supplementary material for: Persistent Hepatitis B Viral Replication in a FVB/N Mouse Model: Impact of Host and Viral Factors
Source: PLoS One. 2012 May 16;7(5):e36984. doi: 10.1371/journal.pone.0036984 (PMC3353969; doi:10.1371/journal.pone.0036984)
Supplement: Table S1 — The sequences of primers used for construction of HBV replicons, site-directed mutagenesis, and real-time PCR analysis. (DOC) [file pone.0036984.s007.doc]

**Table S1. The sequences of primers used for construction of HBV replicons, site-directed mutagenesis, and real-time PCR analysis**

| Primer | Sequences (5’→ 3’) |
| --- | --- |
| P1 | CCGGAAAGCTTGAGCTCTTCTTTTTCACCTCTGCCTAATCA |
| P2 | CCGGAAAGCTTGAGCTCTTCAAAAAGTTGCATGGTGCTGG |
| A1 | CCCAAGCTTCTATTGATTGGAAAGTATGTC |
| A2 | GAAAATTGAGAGAAGTCCAC |
| B1 | ACAARAATCCTCACAATACC |
| B2 | GAAGATCTGATAGGGGCATTTGGTGGTC |
| N214S | CGTGGTGGACTTCTCTCAGTTTTCTAGGGGGAACACC |
| H337Y | AGTGAGCCCTGCTCAGAATACTGTCTCTGCCATATCG |
| mouse IFN-γ +968 | CTGCTGATGGGAGGAGATGT |
| mouse IFN-γ -1104 | GGAAGCACCAGGTGTCAAGT |
| mouse TNF-α +498 | CCTCCTGGCCAACGGCATGG |
| mouse TNF-α -636 | TCGGCTGACGGTGTGGGTGA |
| mouse CXCL9 +828 | CTGTCTGTTTGCTGGTGAGCTAGA |
| mouse CXCL9 -940 | TAGCACCATCTCTGAGACACAATG |
| mouse CXCL10 +712 | GTTTACCTGAGCTCTTTTATTTCAGATG |
| mouse CXCL10 -824 | TTTTCAGGTTCCTCTGAGTATCTTGA |
| mouse β-actin +331 | GATCTGGCACCACACCTTCT |
| mouse β-actin -433 | CTTTTCACGGTTGGCCTTAG |
