# Supplementary material for: Persistent Hepatitis B Viral Replication in a FVB/N Mouse Model: Impact of Host and Viral Factors
Source: PLoS One. 2012 May 16;7(5):e36984. doi: 10.1371/journal.pone.0036984 (PMC3353969; doi:10.1371/journal.pone.0036984)
Supplement: Methods S1 — IFN-γ ELISPOT assay. DNA immunization. (DOC) [file pone.0036984.s008.doc]

**Methods S1**

**IFN-γ ELISPOT assay**

IFN-γ ELISPOT assays were performed using the BD mouse IFN-γ ELISPOT set (BD Biosciences) according to the manufacturer’s instructions. Briefly, the BD ELISPOT plates were coated with 5 μg/ml of BD NA/LE purified anti-mouse IFN-γ antibody in PBS overnight at 4˚C. The plates were then blocked with RPMI-1640 containing 10% FBS for 2 h at RT. IHLs or splenocytes (10^5^ ~ 10^6^ cells) were added to each well in triplicate. To detect HBV-specific CTL activity in BALB/c (H-2^d^) or C57BL/6 mice (H-2^b^), the spleen cells or IHLs were stimulated with HBV-specific MHC class I peptides. For the BALB/c mice, 5 μg/ml of each of the following peptides were used: HBcAg_131-139_, AYRPPNAPI, H-2K^d^-restricted; HBsAg_28-39_, IPQSLDSWWTSL, H-2L^d^-restricted; and Pol_140-148_, HYFQTRHYL, H-2K^d^-restricted . For the C57BL/6 mice, 5 μg/ml of HBsAg_190-197_, VWLSVIWM, H-2K^b^-restricted were used . For the FVB/N mice, 10 μg/ml of each of the four peptides were used (L-HBsAg_205-219_-N, SLDSWWTSL**N**FLGGT; L-HBsAg_210-224_-N, WTSL**N**FLGGTPVCLG; L-HBsAg_205-219_-S, SLDSWWTSL**S**FLGGT; L-HBsAg_210-224_-S, WTSL**S**FLGGTPVCLG) to stimulate the IHLs or splenocytes isolated from the FVB/N mice injected or immunized with B6.2S. The letters in bold indicate the 214^th^ residue, which is a Ser and an Asn in the B6.2S and B6.2 clones, respectively. Control wells contained unstimulated cells. Peptide stimulation lasted for 18 h at 37˚C. Thereafter, cells were removed and the wells incubated with 2 μg/ml of biotinylated anti-mouse IFN-γ antibody for 2 h at RT, followed by incubation with streptavidin-HRP for 1 h at RT. Finally, the spots were visualized by adding AEC (3-amino-9-ethyl-carbazole) substrate and then analyzed using the ImmunoSpot series 5 analyzer (Cellular Technology, Shaker Heights, OH).

**DNA Immunization.**

Mice were immunized with an HBsAg-expressing plasmid, pORF-HBs-B6.2 or pORF-HBs-B6.2S. Briefly, after being anesthetized with ketamine and xylazine, the mice were intramuscularly injected with 100 μg of pORF-HBs-B6.2 or -B6.2S in 50 μl of PBS at the gastrocnemius muscle, followed by electroporation (100V, 100 ms/pulse, 6 pulses with a 100 ms interval) to enhance gene delivery efficiency. Three days later, the mice were intramuscularly injected with 100 μg of pB-CpG20 (a kind gift from Dr. K. Okuda, Yokohama City University) in 50 μL of PBS at the same site as an adjuvant. The mice were boosted every two weeks with the same amount of DNA for either three times (FVB/N mice) or once (C57BL/6). One week after the final boost, the mice were sacrificed for ELISPOT analysis.

**References**

1. Chen A, Wang L, Zhang J, Zou L, Jia Z, et al. (2005) H-2 Kd-restricted hepatitis B virus-derived epitope whose specific CD8+ T lymphocytes can produce gamma interferon without cytotoxicity. J Virol 79: 5568-5576.

2. Schirmbeck R, Melber K, Mertens T, Reimann J (1994) Selective stimulation of murine cytotoxic T cell and antibody responses by particulate or monomeric hepatitis B virus surface (S) antigen. Eur J Immunol 24: 1088-1096.

3. Kakimi K, Isogawa M, Chung J, Sette A, Chisari FV (2002) Immunogenicity and tolerogenicity of hepatitis B virus structural and nonstructural proteins: implications for immunotherapy of persistent viral infections. J Virol 76: 8609-8620.

4. Schirmbeck R, Bohm W, Fissolo N, Melber K, Reimann J (2003) Different immunogenicity of H-2 Kb-restricted epitopes in natural variants of the hepatitis B surface antigen. Eur J Immunol 33: 2429-2438.

5. Kojima Y, Xin KQ, Ooki T, Hamajima K, Oikawa T, et al. (2002) Adjuvant effect of multi-CpG motifs on an HIV-1 DNA vaccine. Vaccine 20: 2857-2865.
